# An evolutionary medicine perspective on pain and its disorders

**DOI:** 10.1098/rstb.2019.0288

**Published:** 2019-09-23

**Authors:** Randolph M. Nesse, Jay Schulkin

**Affiliations:** 1 School of Life Sciences, Arizona State University, Tempe, AZ, USA; 2 University of Washington, Seattle, WA, USA

**Keywords:** evolution, pain, natural selection, evolutionary medicine

## Abstract

Enormous progress in understanding the mechanisms that mediate pain can be augmented by an evolutionary medicine perspective on how the capacity for pain gives selective advantages, the trade-offs that shaped the mechanisms, and evolutionary explanations for the system's vulnerability to excessive and chronic pain. Syndromes of deficient pain document tragically the utility of pain to motivate escape from and avoidance of situations causing tissue damage. Much apparently excessive pain is actually normal because the cost of more pain is often vastly less than the cost of too little pain (the smoke detector principle). Vulnerability to pathological pain may be explained in part because natural selection has shaped mechanisms that respond adaptively to repeated tissue damage by decreasing the pain threshold and increasing pain salience. The other half of an evolutionary approach describes the phylogeny of pain mechanisms; the apparent independence of different kinds of pain is of special interest. Painful mental states such as anxiety, guilt and low mood may have evolved from physical pain precursors. Preliminary evidence for this is found in anatomic and genetic data. Such insights from evolutionary medicine may help in understanding vulnerability to chronic pain.

This article is part of the Theo Murphy meeting issue ‘Evolution of mechanisms and behaviour important for pain’.

## 1. Background

Pain always seems like a problem, but usually, it is part of the solution. Sometimes, however, pain is far too intense or long-lasting, causing enormous useless suffering. The vast majority of research into the causes has focused on the mechanisms that mediate and regulate pain. That research has given rise to a rich body of knowledge that describes the mechanisms that mediate and regulate pain at levels from genes to molecules to tissues and organs [1,2]. However, hopes of finding specific molecules or brain loci to explain pain and pain syndromes have not been fulfilled. Many different genes and molecules interact to make pain possible, and most of them are also involved in many other bodily processes. Nervous system and brain pathways involved in pain are only somewhat specific [3,4]. While specific spinal cord pathways mediate pain transmission, many brain regions and circuits are involved. The entire system provides a fine example of how systems shaped by natural selection are characterized by organic complexity that is fundamentally different from the complexity in systems designed by engineers [5].

It is increasingly clear that understanding pain syndromes requires understanding not only the mechanisms that regulate pain, but also the evolutionary reasons why those mechanisms are vulnerable to failure [4]. They fail for many people, often, and tragically. In the USA, 10% of people report chronic severe back pain, the single greatest cause of Years Lived with Disability. The economic cost of pain conditions is greater than that from heart disease and cancer [6]. Most pain seems excessive; as the philosopher Schopenhauer said in 1851, ‘If the immediate and direct purpose of our life is not suffering, then our existence is the most ill-adapted to its purpose in the world’ [7, p. 41]. Eighty years later, Charles Darwin's *The Origin of Species* provides an explanation for why bodily traits are so well suited to their functions, and why pain exists.

Pain or suffering of any kind, if long continued, causes depression and lessens the power of action; yet it is well adapted to make a creature guard itself against any great or sudden evil.

Charles Darwin, 1887, pp. 51–52 [8].

**Figure 1.**
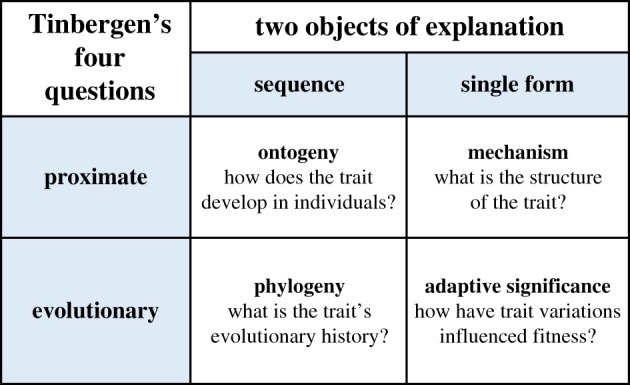
Tinbergen's four questions.

The second half of the twentieth century saw Darwin's ideas applied to animal behaviour. In particular, the Nobel Prize-winning ethologist Nico Tinbergen recognized that four somewhat separate questions must all be answered to provide a complete biological explanation for any trait [9]. Two of them are so-called ‘proximate questions’, one about the structure of a mechanism and the other about how the mechanism develops across the life course of an individual organism. The other two are evolutionary questions. One asks about the adaptive significance of the trait and the selective advantages that shaped it. The other asks about the phylogeny of the trait. Both are relevant to a full understanding of pain. The four questions can be organized into a 2 × 2 table [10] (figure 1).

The fields of behavioural ecology and animal behaviour have prospered thanks to new attention to the ways that genetic variations influence the brain and behavioural variations that influence the prevalence of genetic representations in future generations [11–13]. Recognition that an allele's representation in future generations depends on reproductive success irrespective of lifespan has been a major advance [14,15]. Another is a recognition that selection operates mainly at the level of the gene, and that cooperation among cells in metazoans is superb because all cells are initially genetically identical [16–18].

More recently, it has become clear there are evolutionary explanations not only for why traits work so well but also for why they are vulnerable to failure [19]. For instance, ageing results not only from alleles outside the reach of natural selection but also from antagonistic pleiotropy; alleles that cause ageing are selected for because they give advantages early in life when selection is stronger. Cancer has been suppressed strongly by natural selection, but protection is limited because having more stem cells speeds tissue healing at the cost of cancer vulnerability [20]. Proposing and testing hypotheses for such explanations is a major focus for the new field of evolutionary medicine [21–24]. One well-recognized answer is that natural selection is not all-powerful; mutations happen, the body's mechanisms cannot be perfectly optimized, and they can be damaged by toxins and trauma. Evolutionary medicine augments this observation by encouraging attention to additional possible explanations including mismatch with modern environments, pathogens that evolve faster than their hosts, trade-offs that limit the perfection of every trait, and recognition that natural selection shapes organisms not for longevity, health or freedom from pain, but for maximizing reproduction [19].

We first summarize the evidence for the adaptive value of pain, then describe how natural selection shaped mechanisms that regulate pain expression. This leads naturally to considering the trade-offs arising from deficient versus excessive pain sensitivity, and how the smoke detector principle explains the prevalence of pain that is normal but useless or excessive in the individual instance. We then describe the plasticity mechanisms that adjust pain sensitivity, duration and intensity in response to prior experience, and why those mechanisms increase vulnerability to chronic pain. Finally, we consider the other half of an evolutionary explanation, phylogeny, to describe the unity and diversity of pain mechanisms, and whether capacities for mental pain have evolutionary precursors in physical pain mechanisms.

## 2. How selection shaped the pain system

Substantial individual variation in pain responses has long been clinically obvious, and experimental studies find enormous differences in pain thresholds [25]. Variations in pain sensitivity are heritable, with about 40% of the variation accounted for by genetic variations [26] and specific haplotypes causing two- to threefold differences in pain sensitivity [27]. Heritable variation in pain is present, so natural selection will shape it. The evolutionary question becomes obvious: what advantages and disadvantages are experienced by individuals whose pain threshold is low versus those whose pain threshold is high?

A logically prior question is whether the thresholds for different kinds of pain are highly correlated within individuals. The answer seems to be, somewhat surprisingly, no [28]. Someone who is extremely sensitive to pain from heat may be relatively insensitive to pain from the pressure. This suggests that no single mechanism regulates pain sensitivity in general, and it further suggests that different kinds of nociception diverged long ago or that they had separate origins.

The adaptive value of pain is demonstrated, often tragically, by syndromes of pain deficiency [29]. People born with no capacity for experiencing pain accumulate increasing tissue damage, especially to their skin and joints, and they fail to get full defence against diseases and trauma. The result is deformity, mobility problems, and early death. Notably, damage results not only from exogenous factors but also from lack of motivation for the small movements that protect joints and skin from damage caused by pressure or loss of blood supply; these problems demonstrate the role of nociception in motivating adaptive fidgeting. As some have noted, pain occurs only when the subtle cues of nociception have failed to provide adequate protection [30].

Acquired syndromes of pain deficiency provided further evidence for the value of pain [31]. Patients with syphilis get Charcot's joints because the destruction of pain pathways decreases normal small movements that protect cartilage and bone. Patients with leprosy lose peripheral pain and sensitivity to the extent that smoking cigarettes can result in loss of flesh down to the bone. Patients with central spinal cord lesions likewise experience flesh damage because of insufficient pain.

In addition to protecting against tissue damage, pain also promotes healing by limiting movement and disruption [32]. Pain can also communicate danger and the need for help [33,34].

Compared to syndromes of pain deficiency, those characterized by excessive pain are far more obvious. They are also far more common, for the good evolutionary reason that pain excess harms fitness less than pain deficiency. That does not make such syndromes less of a problem. Patients with hereditary pain syndromes are in constant agony, and millions of people experience chronic back, abdominal, skin or head pain syndromes that can be exceedingly difficult to treat. Why did not natural selection provide better protection against such suffering? Several reasons will be considered in turn, starting with pain that is normal but useless or excessive in the individual instance.

## 3. The smoke detector principle

More often than not, pain seems to be excessive in intensity or duration. Evidence to augment the testimony of individual subjective experience is provided by the safety of analgesics in many instances. This poses an evolutionary mystery. Why would natural selection shape a regulation mechanism that expresses pain when it is not needed? More generally, why are defences such as cough, fatigue, vomiting, anxiety and inflammation so often excessive as demonstrated by the apparent safety of drugs that block them?

One part of the answer comes from the smoke detector principle [35]. If the magnitude of a threat is uncertain, what response threshold will maximize fitness? The answer depends on the cost of the defensive response and the cost of not expressing the response if the danger is actually present. Many responses, especially behavioural responses to physical danger, or inflammatory responses to infections, are relatively inexpensive compared to the catastrophe that could result from an inadequate response. In technical terms, natural selection shapes mechanisms that regulate defences based on the principles of signal detection, the mathematical theory that describes the costs and benefits of responding or not responding in situations of uncertainty [36]. Technical treatments are available elsewhere, but they can be summarized by noting that false alarms and apparently excessive responses are prevalent in the body, just as they are in home smoke detectors. This is a major part of the explanation for why analgesics can often be used safely.

However, excessive analgesia causes major problems, nonetheless. For instance, patients with osteoarthritis treated with anti-inflammatory drugs tend to damage their joints and experience faster joint deterioration [37]. More generally, the problems that arise from using drugs that artificially boost pleasure and block pain encourages increased respect for the value of pain [38].

## 4. Sensitization

The responses of many systems are adjusted as a function of experience. Learning by conditioning is a very general example, but other systems also adapt after experience [39]. Sometimes, as in callous formation after mild skin abrasion, this decreases sensitivity. However, repeated arousal of a defensive system can indicate inadequate protection and a situation in which increased the sensitivity of the system may offer benefits greater than the costs.

The evolutionary question is whether such adjustments are products of an adaptive adjustment, or if they are unfortunate side effects. For epilepsy, the phenomenon of seizures increasing the rate of future seizures (kindling) is a maladaptive by-product [40]. The tendency of depressive episodes to make future episodes more likely has also been seen as pathological [41], however, repeated failures that arouses low mood might well indicate an unrewarding environment in which earlier disengagement of effort is wise [42]. By contrast, increased sensitivity to cues of danger after repeated experiences of pain seems well adapted to provide needed extra protection [43,44].

Sensitization to pain is well recognized [45]. In the immediate aftermath of tissue damage, local sensitization functions effectively to minimize movement and other disruption that would impair healing. The mechanisms that mediate such sensitization have been the object of intense study because of their obvious relevance to chronic pain. In particular, at least 28 transient receptor ion channels have been identified, classified into six subfamilies that respond to different changes, especially temperature [46]. Their activation influences sensitization.

Even more important for understanding chronic pain are the mechanisms that turn off the sensitization associated with tissue healing. What cues are involved? What mechanisms transmit the signal that sensitization is no longer necessary? Can analgesics disrupt the signals that normally desensitize the system? Research on such questions may be a valuable complement to studies of how sensitization is turned on.

This normal sensitization and desensitization process may or may not mediate the sensitization that can result from repeated arousal. Such facultative adaptations that decrease response threshold after repeated arousal are inherently vulnerable to runaway positive feedback because lowering the threshold makes arousal more likely. We speculate that this risk has shaped additional systems to protect against such runaway positive feedback dysregulation and that failures of such systems, or their disruption by drugs, could be implicated in chronic pain syndromes.

## 5. Mismatch

Many diseases are more common for individuals living in modern environments, including atherosclerosis and autoimmune diseases [22,47]. Back and joint pain are common everywhere but have been thought to be increased by sedentary lifestyles and sitting instead of walking and standing. The effects of everyday injuries have received less attention. Modern lives are remarkably free from the cuts and bruises our ancestors experienced routinely. Does this change pain sensitivity? Our ancestors also did not have access to anti-inflammatory and analgesic drugs that are now administered routinely. A recent study shows post-operative pain is prolonged by repeated opioid administration in rats [48]. Is this an example of the kind of receptor changes routinely observed in response to drugs, or something special? The vastly increased number of menstrual cycles for modern versus ancestral women has been suggested to account for the prevalence of chronic pelvic pain and its association with dysmenorrhea [49]. Studies of chronic and other pain in hunter–gatherer populations would provide very valuable data to assess the influence of modern environments on pain experience and the risk of chronic pain.

## 6. The stress response system and pain

The stress response system adjusts bodily systems for action in the face of threat and opportunity [50]. It is a product of millions of years of selection shaping the response itself and the mechanisms that regulate it. The system is usually turned off because of its costs, including decreased immune response, increased metabolic demand and generation of harmful products [51]. But when action is needed, these costs are worth it. The maximum benefit comes from subtle regulation of corticotropin releasing hormone (CRH), adrenocorticotropic hormone, and cortisol. The feedback systems are intricately connected at all levels, with responses not only to hormone levels but also to rates of change [52].

The connection to the pain system is especially obvious in the proopiomelanocortin molecule, which contains the precursors for corticotrophin-releasing hormone (CRF) as well as endorphins. Endorphins decrease pain sensitivity in the face of severe danger when action is essential despite tissue damage [53]. CRF not only initiates the hypothalamic–pituitary–adrenal axis (HPA) axis of the acute stress response, it also increases anxiety and the pain threshold via related chemical signalling systems [54,55]. Decreased sensitivity to pressure pain inducted by CRH injection is not reversed by opioid blockers [56]. Direct administration of hydrocortisone and dexamethasone do not influence the pain threshold, but the threshold is low for individuals whose baseline cortisol is high [57]. Chronic opioid use can disrupt HPA signalling resulting in adrenal insufficiency in up to 25% of cases [58].

Cortisol is often thought of as a stress hormone, but it adjusts the body to opportunities as well as threats that change needs for energy metabolism [59,60]. It is not a direct cause of tissue damage, and its role in decreasing inflammation seems to protect against damage caused by other aspects of the emergency response [61]. Psychological stress can activate the HPA system, but it is by no means consistent, with no cortisol response in about a third of people who take the Trier Social Stress test [62]. Exercise, sudden opportunities and novelty are also potent but somewhat inconsistent stimulators of the system [63–66].

The HPA system is often assumed to be useful in the short term, but costly if aroused for extended periods [67,68]. What is essential is turning cortisol on when it is needed and turning it off when it is not. There certainly are costs from extended arousal, including tissue damage, atherosclerotic progression and possibly chronic pain [69]. The question is whether these costs are worth it on average, or if they are pathological products of a system that is poorly regulated or exposed to novel environments deficient in physical activity that would minimize the impact of stress associated tissue damage.

To sum up, it is obvious and extensively documented that the pain system is a useful adaptation shaped by natural selection. What is newer and still in need of documentation are the reasons why the system so often gives rise to useless pain. The smoke detector principle is an important explanation for some pain and fear that is normal but useless in the specific instance. The sensitization of mechanisms that detect damage is an adaptation in the short run to facilitate tissue healing, and it may also be useful in the long run to provide extra protection in environments that are especially dangerous. However, such protection comes at the risk of the system going into a positive feedback loop in which the experience of pain lowers the pain threshold to cause chronic pain. Mismatch with modern environments may also play important roles in chronic pain, via routes as varied as the effects of exercise, the use of analgesics, and even the frequency of menstrual cycling.

## 7. How are psychic and physical pain related?

Many emotions, such as anxiety, jealousy, envy and boredom, are aversive. That aversiveness contributes to their function by motivating escape and avoidance of situations that harm fitness. Older attempts to describe the evolution of emotions in terms of specific functions for each one are being replaced by the recognition that each emotion is a special mode of response readiness that adjusts many parameters to increase the ability to cope with a situation that has recurred over evolutionary time [70–72].

Depression is a kind of pain and such psychic pain serves functions similar to physical pain [73–75]. Escaping ‘psychache’ has been hypothesized to be the crucial common factor motivating suicide [75–77]. Low mood, that is mild depressive symptoms aroused for a good reason, can be useful in situations where the action is useless or harmful and waiting or withdrawing is more useful. Such situations include infection, losing a status competition, and failing efforts to reach a goal [42].

Substantial evidence supports the role of inflammation and infection in arousing negative affect [78]. The use of sickness behaviour has been recognized ever since the pioneering articles by Benjamin Hart [79]. During an infection, decreasing motivation and activity conserves resources that can be allocated to expensive immune responses, and it also reduces exposure to threats and competitions that are likely to go badly in a depleted state.

Further evidence for the role of infection is provided by the prevalence of depression in patients receiving interferon treatment. Up to a third develops severe depression, with pessimism and self-blame and even suicidal thoughts, a combination of symptoms suggesting that social factors are integrated into the response [80].

Chronic fatigue syndrome is often characterized by aches and pains and general pain sensitivity. While no specific mechanism has yet been found, it seems likely it is related to inappropriate arousal of the sickness behaviour system [79,81,82]. The associated low mood and anxiety provide further support for a close connection among these systems.

There are many other situations in which depressive symptoms may be useful. A large body of work shows that failing efforts arouse low mood, which then motivates waiting, changing strategies or disengaging from the goal [83–87]. The decision to disengage is, however, problematic when substantial resources have already been invested and no good alternative route to the goal is available. This helps to explain why so many people find themselves trapped pursuing unreachable goals despite escalating depression; the costs and risks of stopping are just too great [42].

Situations that involve exclusion or threatened exclusion from a group also arouse psychic pain, as John Bowby suggested with his original studies of attachment [88]. Many others have supported the possible value of psychic pain to prevent such losses [89,90]. Extraordinary sensitivity to one's perceived value to a group influences self-esteem, and low self-esteem can motivate sacrifices that benefit the group [91–93]. Closely related, the pain of grief has been considered as useful or as an epiphenomenon of attachment [94,95].

The relationship between depression and pain has been the object of many studies [96–98]. Patients with chronic pain are especially likely to become depressed. This is in part because they cannot participate fully in life, but it appears that direct connections from depression to pain sensitivity may also be involved. Possible antidepressant effects of anti-inflammatory drugs are being investigated [99]. Conversely, depression can increase pain sensitivity, and brain variations that predispose to chronic pain are not in pain-mediating regions *per se*, but in corticothalamic pathways [100]. The pair of phenomena risks entering a positive feedback loop, especially if opiates have been used to try to decrease the pain.

Brain mechanisms mediating physical pain [1] have been found to have close relationships to mechanisms mediating psychic pain [73,89,101]. A meta-analysis of 18 studies found substantial variation in brain regions involved in various kinds of psychic pain, with no one brain region reliably active in all studies, but intriguing overlap nonetheless with regions associated with physical pain [101]. As far as we can tell, it is not yet known if genetic evidence supports the evolution of psychic pain mechanisms from those that mediate physical pain, but this might well be a valuable topic of study.

## 8. Conclusion

The general conclusion that the capacity for pain is an adaptation shaped by natural selection is not new or controversial. What evolutionary medicine adds is attention to three possible reasons why the system is vulnerable to dysregulation. The smoke detector principle helps to explain apparently excessive responses that can be normal in the face of uncertain threats; systems that adapt to repeated arousal by decreasing thresholds are inherently vulnerable to runaway positive feedback; and, the likely shared evolutionary origins of physical and psychic pain help to provide a context for understanding aversive emotions and their connections to chronic pain. Systematic assessment of pain from an evolutionary perspective has just begun. More and better answers to the questions outlined above may prove clinically useful.

## Data accessibility

This article has no additional data.

## References

[1] Apkarian AV, Bushnell MC, Treede R-D, Zubieta J-K 2005 Human brain mechanisms of pain perception and regulation in health and disease. Eur. J. Pain. 9, 463 (10.1016/j.ejpain.2004.11.001) 15979027

[2] Basbaum AI, Bautista DM, Scherrer G, Julius D 2009 Cellular and molecular mechanisms of pain. Cell 139, 267–284. (10.1016/j.cell.2009.09.028) 19837031 PMC2852643

[3] Walters ET, Moroz LL 2009 Molluscan memory of injury: evolutionary insights into chronic pain and neurological disorders. Brain Behav. Evol. 74, 206–218. (10.1159/000258667) 20029184 PMC2855280

[4] de C Williams AC 2016 What can evolutionary theory tell us about chronic pain? Pain 157, 788–790. (10.1097/j.pain.0000000000000464) 26683235

[5] Swanson LW 2012 Brain architecture: understanding the basic plan, 2nd edn New York, NY: Oxford University Press.

[6] Henschke N, Kamper SJ, Maher CG 2015 The epidemiology and economic consequences of pain. Mayo Clin Proc. 90, 139–147. (10.1016/j.mayocp.2014.09.010) 25572198

[7] Schopenhauer A, Hollingdale RJ 1970 Essays and aphorisms. Harmondsworth, UK: Penguin Books.

[8] Darwin C, Darwin F 1887 The life and letters of Charles Darwin, including an autobiographical chapter, 3d edn London, UK: J. Murray.

[9] Tinbergen N 1963 On the aims and methods of ethology. Z. Für. Tierpsychol. 20, 410–463. (10.1111/j.1439-0310.1963.tb01161.x)

[10] Nesse RM 2013 Tinbergen's four questions, organized: a response to Bateson and Laland. Trends Ecol. Evol. 28, 681–682. (10.1016/j.tree.2013.10.008) 24216179

[11] Alcock J 2013 Animal behavior : an evolutionary approach, 10th edn Sunderland, MA: Sinauer Associates.

[12] Alcock J, Sherman P 2010 The utility of the proximate-ultimate dichotomy in ethology. Ethology 96, 58–62. (10.1111/j.1439-0310.1994.tb00881.x)

[13] Westneat DF, Fox CW 2010 Evolutionary behavioral ecology. Oxford, NY: Oxford University Press.

[14] de C Williams AC 2001 Pleiotropy, natural selection, and the evolution of senescence. Sci. SAGE KE 2001, 13.

[15] Gaillard J-M, Lemaître J-F 2017 The Williams' legacy: a critical reappraisal of his nine predictions about the evolution of senescence: THE WILLIAMS’ LEGACY. Evolution 71, 2768–2785. (10.1111/evo.13379) 29053173

[16] Alcock J 2017 Human sociobiology and group selection theory. In On human nature, pp. 383–396. Elsevier (cited 20 Jun 2019). See https://linkinghub.elsevier.com/retrieve/pii/B9780124201903000235

[17] West SA, Griffin AS, Gardner A 2007 Social semantics: altruism, cooperation, mutualism, strong reciprocity and group selection. J. Evol. Biol. 20, 415–432. (10.1111/j.1420-9101.2006.01258.x) 17305808

[18] Williams GC 1966 Adaptation and natural selection: a critique of some current evolutionary thought. Princeton, NJ: Princeton University Press.

[19] Nesse RM 2005 Maladaptation and natural selection. Q. Rev. Biol. 80, 62–70. (10.1086/431026) 15884737

[20] Greaves M 2010 Cancer stem cells: back to Darwin? Semin. Cancer Biol. 20, 65–70. (10.1016/j.semcancer.2010.03.002) 20359535

[21] Gluckman P, Beedle A, Hanson M 2009 Principles of evolutionary medicine. Oxford, UK: Oxford University Press.

[22] Nesse RM, Williams GC 1994 Why we get sick: the New science of Darwinian medicine. New York, NY: Vintage Books.

[23] Perlman R 2013 Evolution and medicine. Oxford, UK: Oxford University Press.

[24] Stearns SC 2012 Evolutionary medicine: its scope, interest and potential. Proc. Biol. Sci. 279, 4305–4321. (10.1098/rspb.2012.1326) 22933370 PMC3479795

[25] Nielsen CS, Staud R, Price DD 2009 Individual differences in pain sensitivity: measurement, causation, and consequences. J. Pain 10, 231–237. (10.1016/j.jpain.2008.09.010) 19185545

[26] Mogil JS 2012 Pain genetics: past, present and future. Trends Genet. 28, 258–266. (10.1016/j.tig.2012.02.004) 22464640

[27] Diatchenko L et al. 2005 Genetic basis for individual variations in pain perception and the development of a chronic pain condition. Hum. Mol. Genet. 14, 135–143. (10.1093/hmg/ddi013) 15537663

[28] Diatchenko L, Nackley AG, Tchivileva IE, Shabalina SA, Maixner W 2007 Genetic architecture of human pain perception. Trends Genet. 23, 605–613. (10.1016/j.tig.2007.09.004) 18023497

[29] Nagasako EM, Oaklander AL, Dworkin RH 2003 Congenital insensitivity to pain: an update. Pain 101, 213–219. (10.1016/S0304-3959(02)00482-7) 12583863

[30] Baliki MN, Apkarian AV 2015 Nociception, pain, negative moods, and behavior selection. Neuron 87, 474–491. (10.1016/j.neuron.2015.06.005) 26247858 PMC4529956

[31] Alpert SW, Koval KJ, Zuckerman JD 1996 Neuropathic arthropathy: review of current knowledge. J. Am. Acad. Orthop. Surg. 4, 100–108. (10.5435/00124635-199603000-00005) 10795044

[32] Wall PD, Melzack R 1996 The challenge of pain. Harmondsworth, UK: Penguin.

[33] Steinkopf L 2016 An evolutionary perspective on pain communication. Evol. Psychol. 14, 147470491665396 (10.1177/1474704916653964)

[34] de C Williams AC 2002 Facial expression of pain: an evolutionary account. Behav. Brain Sci. 25, 439–455. 12879700 10.1017/s0140525x02000080

[35] Nesse RM 2005 Natural selection and the regulation of defenses: a signal detection analysis of the smoke detector principle. Evol. Hum. Behav. 26, 88–105. (10.1016/j.evolhumbehav.2004.08.002)

[36] Green DM, Swets JA 1966 Signal detection theory and psycho-physics. New York, NY: Wiley.

[37] Huskisson EC, Berry H, Gishen P, Jubb RW, Whitehead J 1995 Effects of antiinflammatory drugs on the progression of osteoarthritis of the knee. LINK Study Group. Longitudinal investigation of nonsteroidal antiinflammatory drugs in knee osteoarthritis. J. Rheumatol. 22, 1941–1946. 8991995

[38] Linden DJ 2012 The compass of pleasure: how our brains make fatty foods, orgasm, exercise, marijuana, generosity, vodka, learning, and gambling feel so good, 242 p Harmondsworth, UK: Penguin.

[39] Rosen JB, Schulkin J 1998 From normal fear to pathological anxiety. Psychol. Rev. 105, 325–350. (10.1037/0033-295X.105.2.325) 9577241

[40] Morimoto K, Fahnestock M, Racine RJ 2004 Kindling and status epilepticus models of epilepsy: rewiring the brain. Prog. Neurobiol. 73, 1–60. (10.1016/j.pneurobio.2004.03.009) 15193778

[41] Monroe SM, Harkness KL 2005 Life stress, the ‘Kindling’ hypothesis, and the recurrence of depression: considerations from a life stress perspective. Psychol. Rev. 112, 417–445. (10.1037/0033-295X.112.2.417) 15783292

[42] Nesse RM 2019 Good reasons for bad feelings: insights from the frontier of evolutionary psychiatry. New York, NY: Dutton Books.

[43] Meacham F, Bergstrom CT 2016 Adaptive behavior can produce maladaptive anxiety due to individual differences in experience. Evol. Med. Public Health 2016, 270–285. (10.1093/emph/eow024) 27530544 PMC5490257

[44] Stein DJ, Nesse RM 2011 Threat detection, precautionary responses, and anxiety disorders. Neurosci. Biobehav. Rev. 35, 1075–1079. (10.1016/j.neubiorev.2010.11.012) 21147162

[45] Woolf CJ 2011 Central sensitization: implications for the diagnosis and treatment of pain. Pain 152(3, Supplement), S2–15. (10.1016/j.pain.2010.09.030) 20961685 PMC3268359

[46] Wang H, Woolf CJ 2005 Pain TRPs. Neuron 46, 9–12. (10.1016/j.neuron.2005.03.011) 15820689

[47] Gluckman PD, Hanson M 2006 Mismatch: why our world no longer fits our bodies. New York, NY: Oxford University Press.

[48] Grace PM, Galer EL, Strand KA, Corrigan K, Berkelhammer D, Maier SF, Watkins LR 2019 Repeated morphine prolongs postoperative pain in male rats. Anesth Analg. 128, 161–167. (10.1213/ane.0000000000003345) 29596097 PMC7054903

[49] Jarrell J, Arendt-Nielsen L 2016 Evolutionary considerations in the development of chronic pelvic pain. Am. J. Obstet. Gynecol. 215, 201.e1–201.e4. (10.1016/j.ajog.2016.05.019) 27269450

[50] Nesse RM, Bhatnagar S, Ellis B 2016 Evolutionary origins and functions of the stress response system. In Stress: concepts, cognition, emotion, and behavior (ed. Fink G), pp. 95–101. Amsterdam, The Netherlands: Elsevier (cited 20 Jun 2019). See https://linkinghub.elsevier.com/retrieve/pii/B978012800951200011X

[51] Schulkin J 2017 The CRF signal: uncovering an information molecule. New York, NY: Oxford University Press.

[52] Young EA, Abelson J, Lightman SL 2004 Cortisol pulsatility and its role in stress regulation and health. Front. Neuroendocrinol. 25, 69–76. (10.1016/j.yfrne.2004.07.001) 15571755

[53] François A et al. 2017 A brainstem-spinal cord inhibitory circuit for mechanical pain modulation by GABA and enkephalins. Neuron 93, 822–839.e6 (10.1016/j.neuron.2017.01.008) 28162807 PMC7354674

[54] Koob GF, Le Moal M 2006 Neurobiology of addiction. Amsterdam, Boston, MA: Elsevier/Academic Press.

[55] Koob GF, Moal ML 2005 Plasticity of reward neurocircuitry and the ‘dark side’ of drug addiction. Nat. Neurosci. 8, 1442 (10.1038/nn1105-1442) 16251985

[56] Matejec R, Uhlich H, Hotz C, Mühling J, Harbach H-W, Bödeker R-H, Hempelmann G, Teschemacher H 2005 Corticotropin-releasing hormone reduces pressure pain sensitivity in humans without involvement of β-endorphin(1–31), but does not reduce heat pain sensitivity. Neuroendocrinology 82, 185–197. (10.1159/000091980) 16534240

[57] Wingenfeld K, Wolf S, Kunz M, Krieg J-C, Lautenbacher S 2015 No effects of hydrocortisone and dexamethasone on pain sensitivity in healthy individuals. Eur. J. Pain 19, 834–841. (10.1002/ejp.610) 25380413

[58] Donegan D, Bancos I 2018 Opioid induced adrenal insufficiency. Mayo Clin Proc. 93, 937–944. (10.1016/j.mayocp.2018.04.010) 29976376

[59] Peciña S, Schulkin J, Berridge KC 2006 Nucleus accumbens corticotropin-releasing factor increases cue-triggered motivation for sucrose reward: paradoxical positive incentive effects in stress? BMC Biol. 4, 8 (10.1186/1741-7007-4-8) 16613600 PMC1459217

[60] Peters A, McEwen BS, Friston K 2017 Uncertainty and stress: why it causes diseases and how it is mastered by the brain. Prog. Neurobiol. 156, 164–188. (10.1016/j.pneurobio.2017.05.004) 28576664

[61] Munck A, Narayfejestoth A 1994 Glucocorticoids and stress-permissive and suppressive actions. Ann. N. Y. Acad. Sci. 746, 115–130. (10.1111/j.1749-6632.1994.tb39221.x) 7825870

[62] Kirschbaum C, Pirke KM, Hellhammer DH 1993 The ‘Trier Social Stress Test’—a tool for investigating psychobiological stress responses in a laboratory setting. Neuropsychobiology 28, 76–81. (10.1159/000119004) 8255414

[63] Baumgartner HM, Schulkin J, Berridge KC 2018 Optogenetic excitation of limbic corticotropin releasing factor neurons modulates motivation. (cited 15 May 2019). See https://www.abstractsonline.com/pp8/#!/4649/presentation/29954

[64] Curtis GC, Buxton M, Lippman D, Nesse RM, Wright J 1976 ‘Flooding *in vivo*’ during the circadian phase of minimal cortisol secretion: anxiety and therapeutic success without adrenal cortical activation. Biol. Psychiat. 11, 101–107. 1260072

[65] Hill EE, Zack E, Battaglini C, Viru M, Viru A, Hackney AC 2008 Exercise and circulating cortisol levels: the intensity threshold effect. J. Endocrinol. Invest. 31, 587–591. (10.1007/BF03345606) 18787373

[66] Tran L, Schulkin J, Greenwood-Van Meerveld B 2014 Importance of CRF receptor-mediated mechanisms of the bed nucleus of the stria terminalis in the processing of anxiety and pain. Neuropsychopharmacology 39, 2633–2645. (10.1038/npp.2014.117) 24853772 PMC4207343

[67] McEwen BS, Bowles NP, Gray JD, Hill MN, Hunter RG, Karatsoreos IN, Nasca C 2015 Mechanisms of stress in the brain. Nat. Neurosci. 18, 1353–1363. (10.1038/nn.4086) 26404710 PMC4933289

[68] Sapolsky RM 2004 Why zebras don't get ulcers: an updated guide to stress, stress-related diseases, and coping, 2nd edn New York, NY: W. F. Freeman.

[69] Vachon-Presseau E et al. 2013 The stress model of chronic pain: evidence from basal cortisol and hippocampal structure and function in humans. Brain 136, 815–827. (10.1093/brain/aws371) 23436504

[70] Nesse RM 1990 Evolutionary explanations of emotions. Hum. Nat. 1, 261–289. (10.1007/BF02733986) 24222085

[71] Plutchik R 2003 Emotions and life: perspectives from psychology, biology, and evolution. Washington, DC: American Psychological Association.

[72] Scherer KR 2005 What are emotions? And how can they be measured? Soc. Sci. Inf. 44, 695–729. (10.1177/0539018405058216)

[73] Mee S, Bunney BG, Reist C, Potkin SG, Bunney WE 2006 Psychological pain: a review of evidence. J. Psychiatr. Res. 40, 680–690. (10.1016/j.jpsychires.2006.03.003) 16725157

[74] Nesse RM 2000 Is Depression an adaptation? Arch. Gen. Psychiatry 57, 14 (10.1001/archpsyc.57.1.14) 10632228

[75] Shneidman ES 1993 Suicide as psychache: a clinical approach to self-destructive behavior. Lanham, MD: Jason Aronson.

[76] Klonsky ED, May AM, Saffer BY 2016 Suicide, suicide attempts, and suicidal ideation. Annu. Rev. Clin. Psychol. 12, 307–330. (10.1146/annurev-clinpsy-021815-093204) 26772209

[77] Soper CA 2018 The evolution of suicide. Berlin, Germany: Springer.

[78] Grace PM, Hutchinson MR, Maier SF, Watkins LR 2014 Pathological pain and the neuroimmune interface. Nat. Rev. Immunol. 14, 217–231. (10.1038/nri3621) 24577438 PMC5525062

[79] Johnson RW 2002 The concept of sickness behavior: a brief chronological account of four key discoveries. Vet. Immunol. Immunopathol. 87, 443–450. (10.1016/S0165-2427(02)00069-7) 12072271

[80] Okada F 1995 Interferon-induced depression: just one of Bonhoeffer's exopgene Reaktionstypen or a clue to understanding psychoimmunological aspects of depression? (letter). J. Mol. Med. 73, 99–100. (10.1007/BF00270585) 7542996

[81] Hart BL 1988 Biological basis of the behavior of sick animals. Neurosci. Biobehav. Rev. 12, 123–137. (10.1016/S0149-7634(88)80004-6) 3050629

[82] Shattuck EC, Muehlenbein MP 2015 Human sickness behavior: ultimate and proximate explanations. Am. J. Phys. Anthropol. 157, 1–18. (10.1002/ajpa.22698) 25639499

[83] Carver CS, Johnson SL, Joormann J, Scheier MF 2015 An evolving view of the structure of self-regulation. In Handbook of biobehavioral approaches to self-regulation (eds Gendolla GHE, Tops M, Koole SL), pp. 9–23. New York, NY: Springer (cited 6 Sept 2018). See http://link.springer.com/10.1007/978-1-4939-1236-0_2

[84] Carver CS, Scheier MF 1990 Origins and functions of positive and negative affect: a control-process view. Psychol. Rev. 97, 19–35. (10.1037/0033-295X.97.1.19)

[85] Haase CM, Heckhausen J, Wrosch C 2013 Developmental regulation across the life span: toward a new synthesis. Dev. Psychol. 49, 964–972. (10.1037/a0029231) 22822930

[86] Klinger E 1975 Consequences of commitment to and disengagement from incentives. Psychol. Rev. 82, 1–25. (10.1037/h0076171)

[87] Wrosch C, Scheier MF, Miller GE, Schulz R, Carver CS 2003 Adaptive self-regulation of unattainable goals: goal disengagement, goal reengagement, and subjective well-being. Pers. Soc. Psychol. Bull. 29, 1494–1508. (10.1177/0146167203256921) 15018681

[88] Bowlby J 1979 The making & breaking of affectional bonds. London, UK: Tavistock Publications Limited.

[89] Eisenberger NI 2012 The pain of social disconnection: examining the shared neural underpinnings of physical and social pain. Nat. Rev. Neurosci. 13, 421–434. (10.1038/nrn3231) 22551663

[90] MacDonald G, Leary MR 2005 Why does social exclusion hurt? The relationship between social and physical pain. Psychol. Bull. 131, 202–223. (10.1037/0033-2909.131.2.202) 15740417

[91] Kirkpatrick LA, Ellis BJ 2001 An evolutionary psychological approach to self-esteem: multiple domains and multiple functions. In Blackwell handbook of social psychology: interpersonal processes (eds Hogg MA, Tindale S), pp. 409–436. Oxford, UK: Blackwell (10.1002/9780470998557.ch16)

[92] Leary MR, Baumeister RF 2000 The nature and function of self-esteem: sociometer theory. In Advances in experimental social psychology (ed. Zanna MP), pp. 2–51. San Diego, CA: Academic Press.

[93] Murray SL, Griffin DW, Rose P, Bellavia GM 2003 Calibrating the sociometer: the relational contingencies of self-esteem. J. Pers. Soc. Psychol. 85, 63–84. (10.1037/0022-3514.85.1.63) 12872885

[94] Archer J 2003 The nature of grief: the evolution and psychology of reactions to loss. London, UK: Routledge.

[95] Nesse RM 2005 An evolutionary framework for understanding grief. In Late life widowhood in the United States (eds Carr D, Nesse R, Wortman CB), pp. 195–226. New York, NY: Springer.

[96] Bair MJ, Robinson RL, Katon W, Kroenke K 2003 Depression and pain comorbidity: a literature review. Arch. Intern. Med. 163, 2433 (10.1001/archinte.163.20.2433) 14609780

[97] de C Williams AC 1998 Depression in chronic pain: mistaken models, missed opportunities. Scand. J. Behav. Ther. 27, 61–80. (10.1080/02845719808408497)

[98] Han C, Pae C-U 2015 Pain and depression: a neurobiological perspective of their relationship. Psychiatry Investig. 12, 1–8. (10.4306/pi.2015.12.1.1) PMC4310906 25670939

[99] Miller AH, Raison CL 2016 The role of inflammation in depression: from evolutionary imperative to modern treatment target. Nat. Rev. Immunol. 16, 22–34. (10.1038/nri.2015.5) 26711676 PMC5542678

[100] Vachon-Presseau E et al. 2016 Corticolimbic anatomical characteristics predetermine risk for chronic pain. Brain 139, 1958–1970. (10.1093/brain/aww100) 27190016 PMC4939699

[101] Meerwijk EL, Ford JM, Weiss SJ 2013 Brain regions associated with psychological pain: implications for a neural network and its relationship to physical pain. Brain Imaging Behav. 7, 1–14. (10.1007/s11682-012-9179-y) 22660945

